# Supplementary material for: Integrated analysis of cervical squamous cell carcinoma cohorts from three continents reveals conserved subtypes of prognostic significance
Source: Nat Commun. 2022 Oct 7;13:5818. doi: 10.1038/s41467-022-33544-x (PMC9547055; doi:10.1038/s41467-022-33544-x)
Supplement: Supplementary file 1 — Supplementary Information [file 41467_2022_33544_MOESM1_ESM.pdf]

Chakravarthy, Reddin et al: Supplementary Information

2 **Supplementary Methods**

4 **Supplementary Figure 1:** tSNE clustering by histology in cervical cancer cohorts.

5 **Supplementary Figure 2:** Consensus clustering using ConsensusClusterPlus.

6 **Supplementary Figure 3:** Genes that are both differentially expressed and differentially methylated between C1 and C2 subgroups.

7 **Supplementary Figure 4:** Concordance between gene expression and DNA methylation-derived cluster membership.

8 **Supplementary Figure 5:** Validation SCC cohorts.

9 **Supplementary Figure 6:** Elevation of epithelial mesenchymal transition (EMT) score is evident in C2 tumours.

10 **Supplementary Figure 7:** Mutational signatures of combined HPV+ squamous cervical cancer cohorts.

11 **Supplementary Figure 8:** Increased levels of YAP in tumours with *YAP1* amplification.

12 **Supplementary Figure 9:** Differences in immune microenvironment between SCC subgroups in individual cohorts.

13 **Supplementary Figure 10:** Immune cell ratios by cluster using MethylCIBERSORT estimates.

14 **Supplementary Figure 11:** Comparison of MethylCIBERSORT estimates and immunohistochemistry (IHC)-based scoring.

15 **Supplementary Figure 12:** Upregulation of immune checkpoint genes in C2 SCCs.

Supplementary Methods

CD8 digital histopathology, cervix cancer

Immunohistochemistry

- The CD8-positive cells were stained with DAB and visualized in brown.
- Hematoxylin was used as counterstain, visualizing cellular cytoplasm with a pale bluish color and cell nuclei in a darker bluish nuance.

Imaging

- The CD8-stained sections were digitized using a whole slide scanner, acquiring a 20× magnification (0.46 μm/pixel) (Figure 1, upper left).
- Image size was reduced by 50% for analysis, acquiring a magnification of 0.92 μm/pixel.

Segmentation of CD8-positive cells

- Color deconvolution was performed to separate out the DAB color-channel, containing information about the intensity of brown in the image (Figure 1, upper right).
- The CD8-positive areas were separated by an intensity threshold on the brown color-channel image.
- Binary images were created (Figure 1, lower left).

Quantification

- An area fraction was calculated: the CD8-positive area (Figure 1, lower right) relative to the total tumor area (region of interest, ROI) (Figure 2).

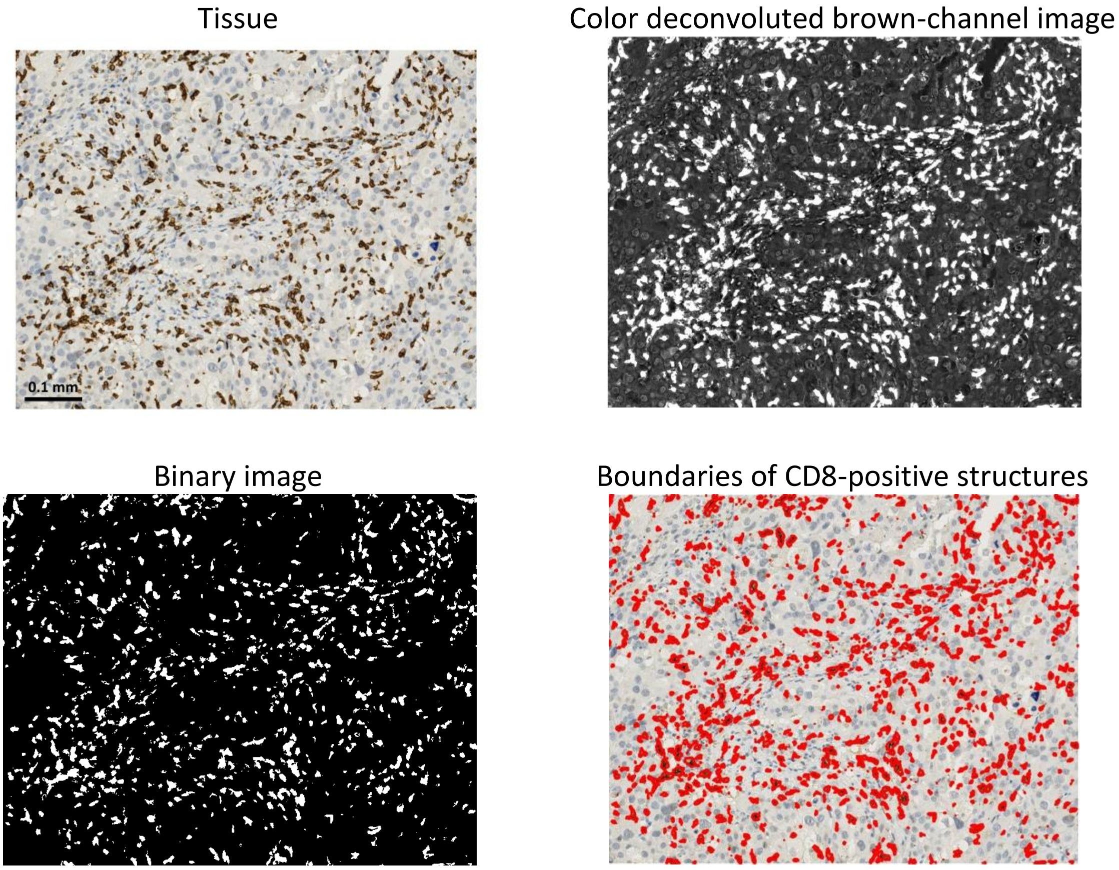

Figure 1: Procedure for segmentation of CD8-positive areas in a CD8-stained biopsy section from a cervix tumor. Scale bar (upper left) indicates 0.1 mm. CD8-positive area is indicated in red (lower, right).

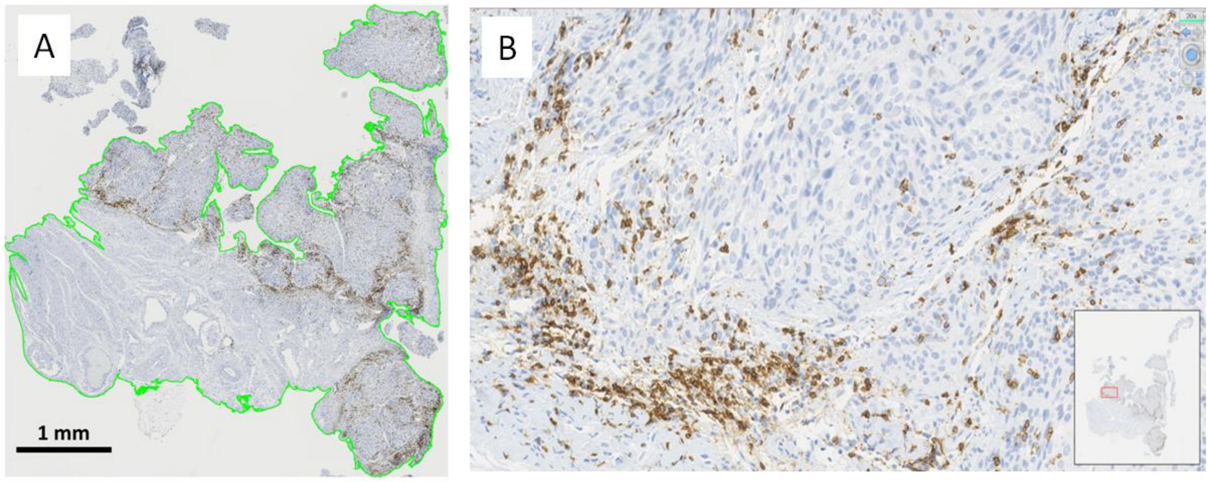

Figure 2: Illustration of the defined region of interest (ROI) in a CD8-stained biopsy section from a cervix tumor. In (A), the ROI used for analysis is outlined in green. Scale bar indicates 1 mm. In (B), part of the section in (A) is shown. Inset shows the section in (A) with the location and size of the presented part indicated in red.

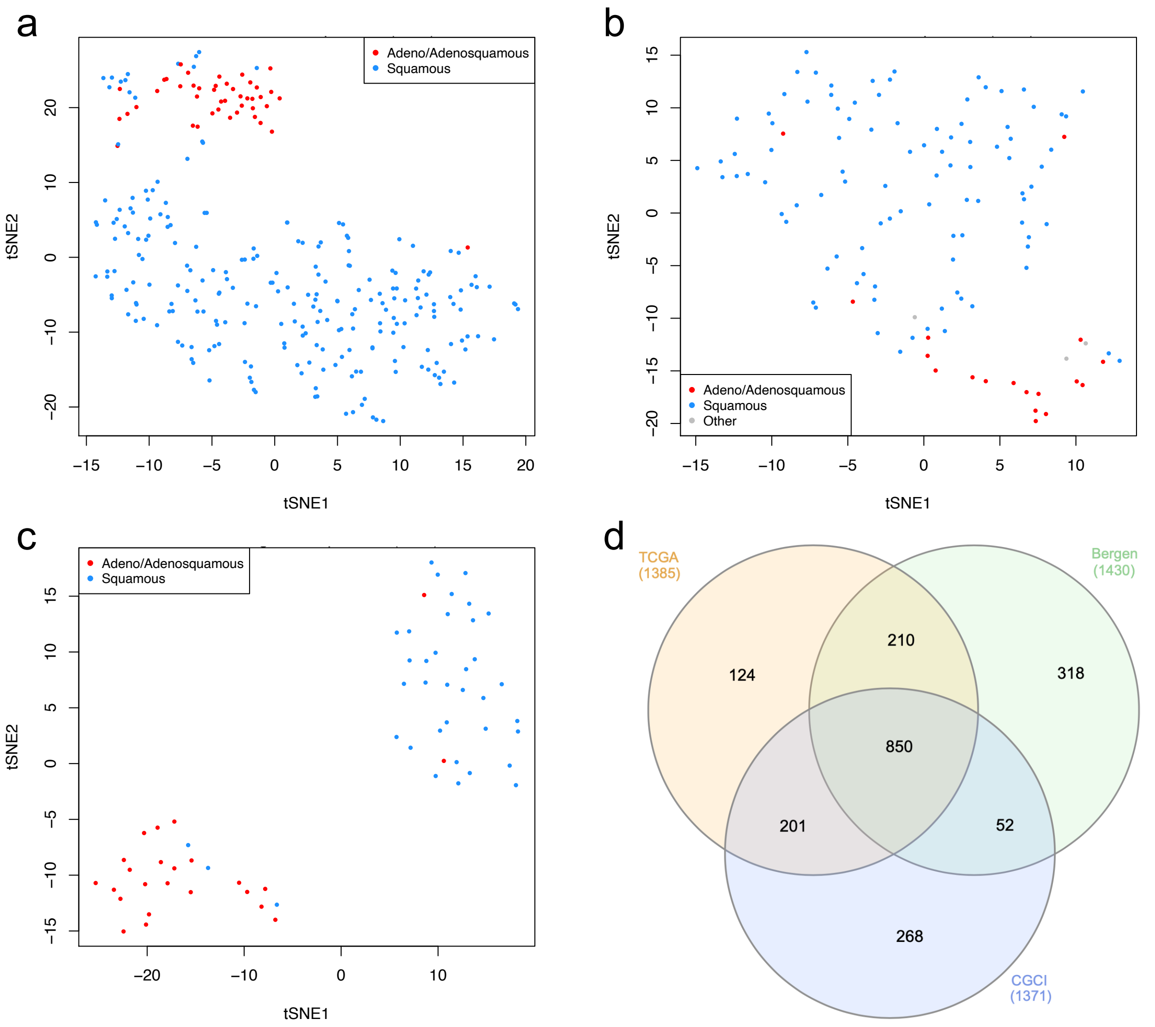

Supplementary Figure 1 – tSNE clustering by histology in cervical cancer cohorts. Unsupervised tSNE analysis using top 10% most variable genes for **a)** TCGA (1385 most variable genes), **b)** Ugandan (1371) and **c)** Bergen (1430) cervical cancer cohorts illustrates molecular differences between squamous and adenocarcinoma/adenosquamous tumours. Concordance of the top 10% most variable genes was high amongst the 3 cohorts **(d)**.

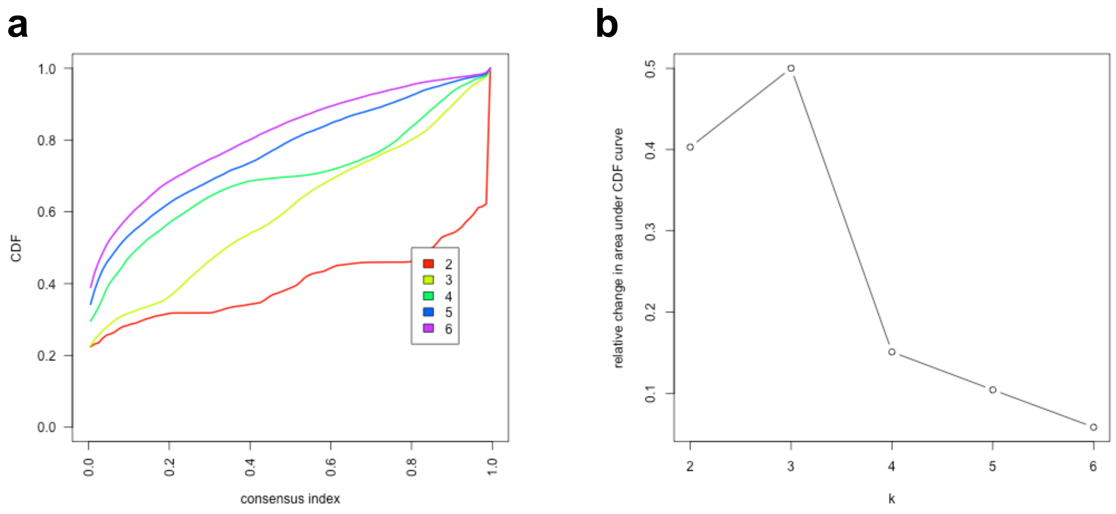

Supplementary Figure 2 Consensus clustering using ConsensusClusterPlus. a) The consensus cumulative distribution function (CDF) plot was used to calculate the proportion of ambiguous clustering (PAC) score which would determine the optimum number of clusters. The PAC score = CDF at 0.9 consensus index - CDF at 0.1 consensus index for each curve. **b)** The delta area plot was also considered used in the decision of optimum number of clusters.

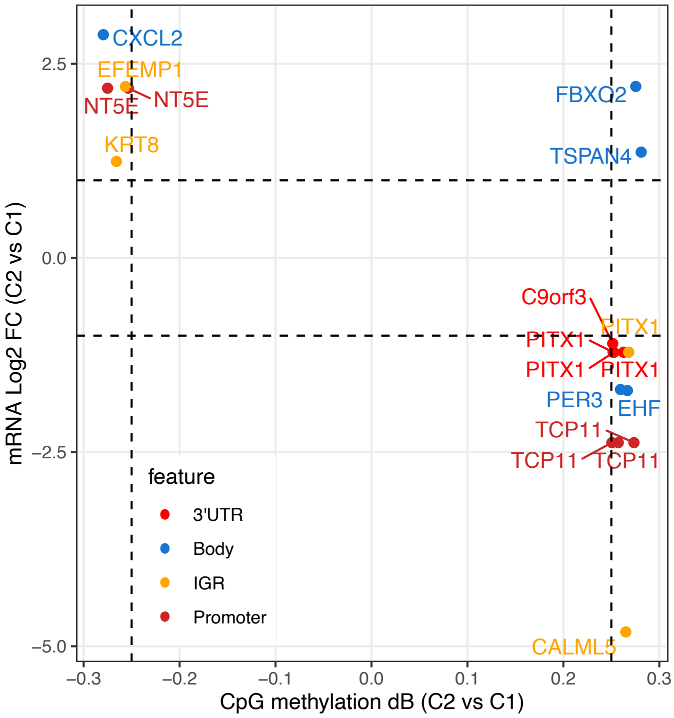

Supplementary Figure 3 Genes that are both differentially expressed and differentially methylated between C1 and C2 subgroups. Datapoints represent methylated variable positions (in either the 3'UTR, body of gene, intergenic region or gene promoter) in genes that are also differentially expressed between C1 and C2 subgroups. Datapoints in the top left quadrant are MVPs that are hypomethylated in genes that are also upregulated in C2 tumours. Those in the bottom right quadrant are hypermethylated in genes that are downregulated in C2 tumours.

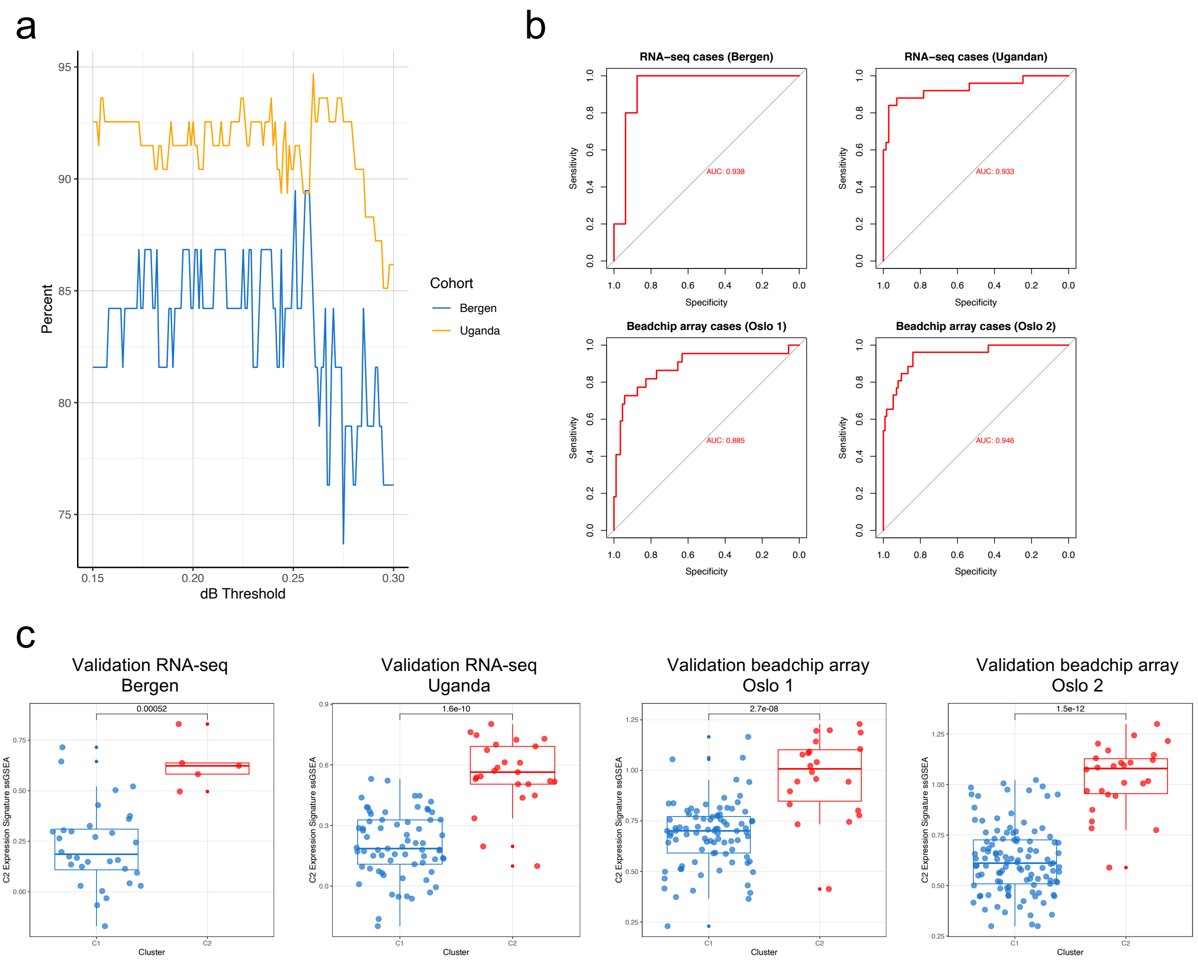

Supplementary Figure 4 Concordance between gene expression and DNA methylation-derived cluster membership. a) The percentage of samples that are designated the same cluster allocation by gene expression signature and methylation signatures based on varying delta Beta thresholds. **b)** ROC curves showing the accuracy with which C1 or C2 cluster membership can be predicted using DNA methylation differences (MVPs) in samples from the validation cohorts for which either RNA-seq (Bergen, n=37, and Uganda, n=94, HPV+ SCC cases), Illumina HumanHT-12 V4.0 expression beadchip array (Oslo SCC cases, n=109) or Illumina HumanWG-6 v3.0 expression beadchip array (Oslo SCC cases, n=139) gene expression data were available. **c)** Single sample gene set enrichment analysis (ssGSEA) for validation cohorts used in panel B. The y-axis represents the ssGSEA score for each sample, compared with the genes from the C2 gene expression signature. The upper line in a box plot represents the upper quartile, the second line the median and the lowest line the lower quartile. The whisker above the box is drawn to the highest point within 1.5x the interquartile range (IQR), the whisker below the box is drawn to the lowest point within 1.5x the IQR. P-values from two-sided Wilcoxon rank-sum test.

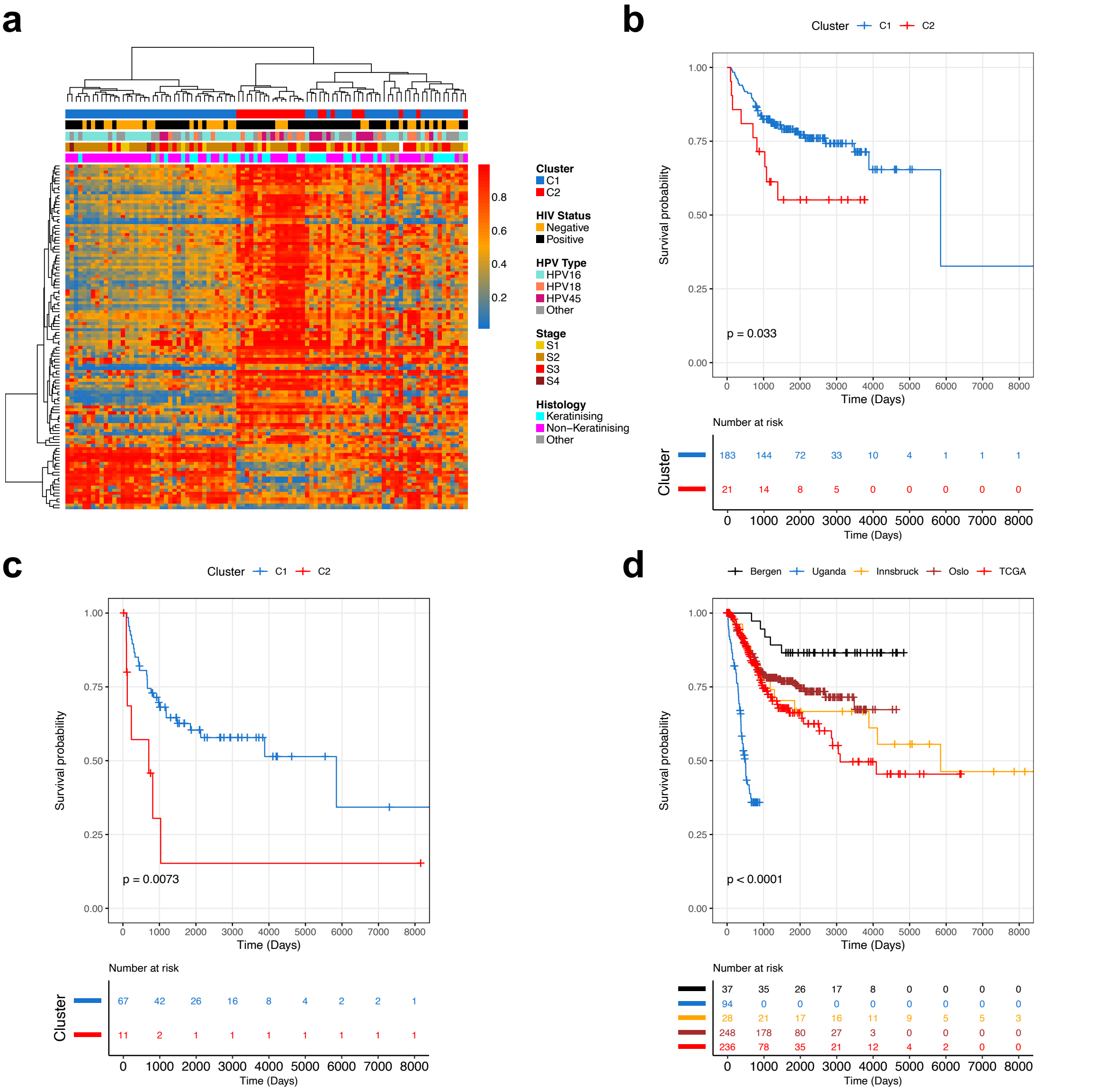

Supplementary Figure 5 Validation SCC cohorts. a) The Ugandan validation cohort clustering based on 116 methylation variable position (MVP) signature. Each row represents one of the 129 MVPs. Kaplan-meier curves for **b)** disease specific survival (DSS) in HPV16+ European validation cohort SCC patients; **c)** DSS for European validation cohort SCC patients without chemotherapy treatment and **d)** survival for the 5 individual cohorts in this study. Statistical test used for Kaplan-meier curves was two-sided log-rank sum test. Source data are provided as a Source Data File.

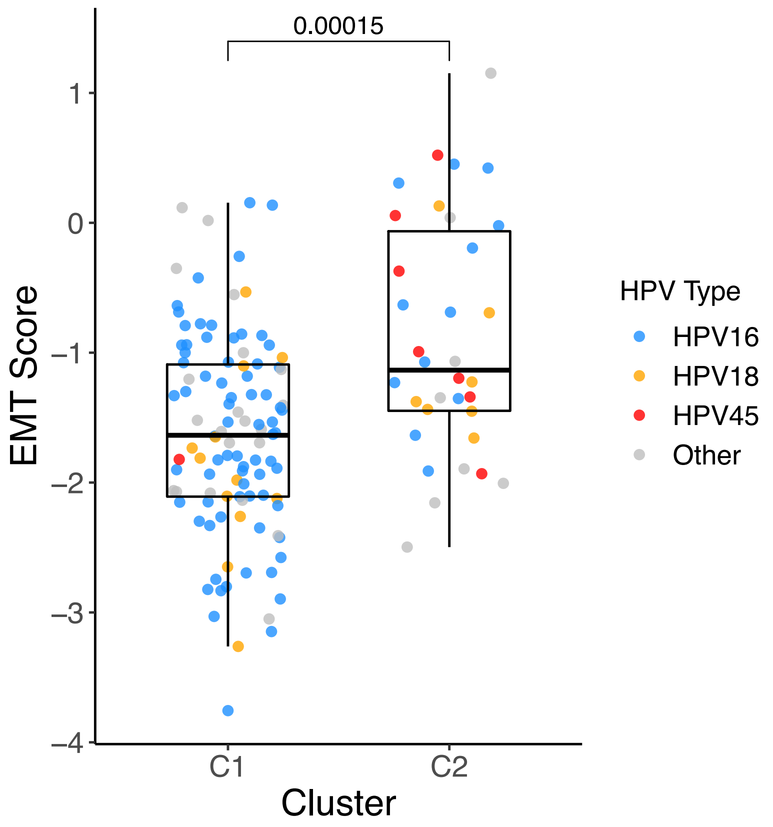

Supplementary Figure 6 Elevation of epithelial mesenchymal transition (EMT) score is evident in C2 tumours. The EMT score derived by TCGA for 140 HPV+ squamous TCGA cervical cancer tumours is higher in the C2 compared to the C1 subgroup in our study. Each point represents a TCGA HPV+ tumour. The upper line in a box plot represents the upper quartile, the second line the median and the lowest line the lower quartile. The whisker above the box is drawn to the highest point within 1.5x the interquartile range (IQR), the whisker below the box is drawn to the lowest point within 1.5x the IQR. The P value is from two-sided Wilcoxon's rank sum test. Source data are provided as a Source Data file.

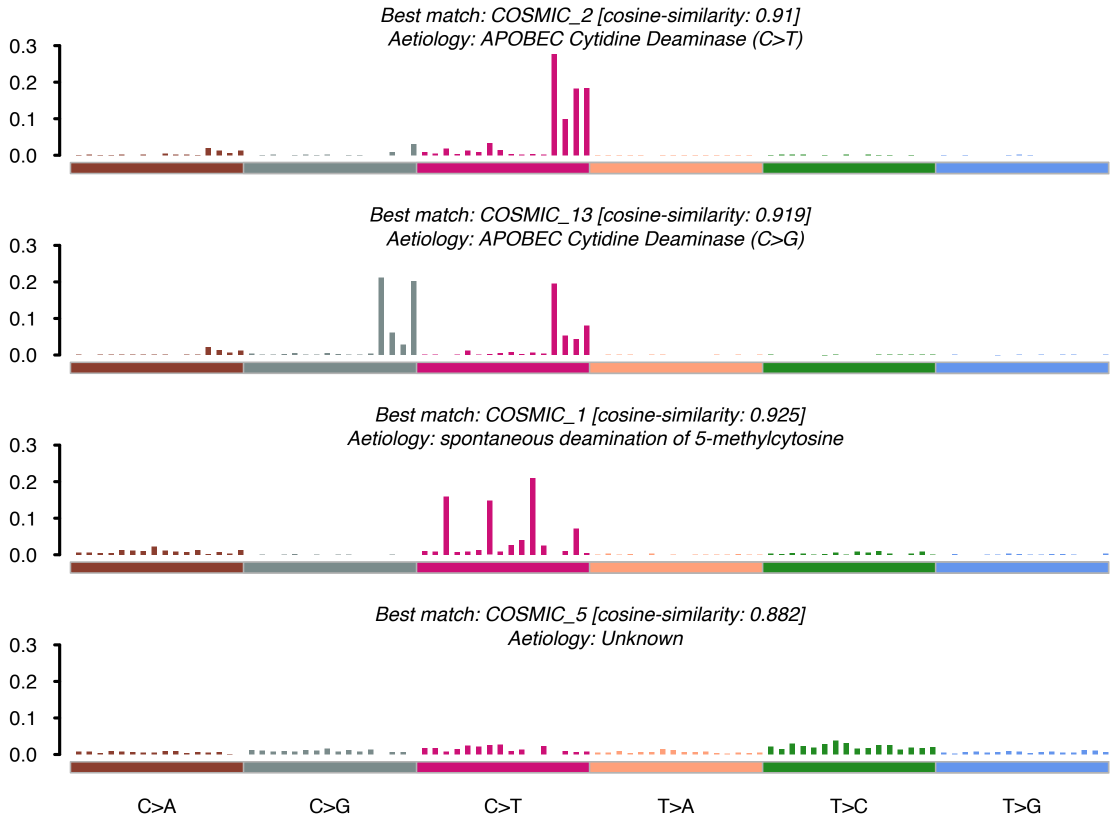

Supplementary Figure 7 Mutational signatures of combined HPV+ squamous cervical cancer cohorts. COSMIC mutational signatures identified in combined HPV+ squamous cervical cancer cohort including genomic data from TCGA, Bergen and Ugandan cohorts. The bars show the proportion of single base substitutions (in 16 different trinucleotide sequences) that contribute to the mutational signature.

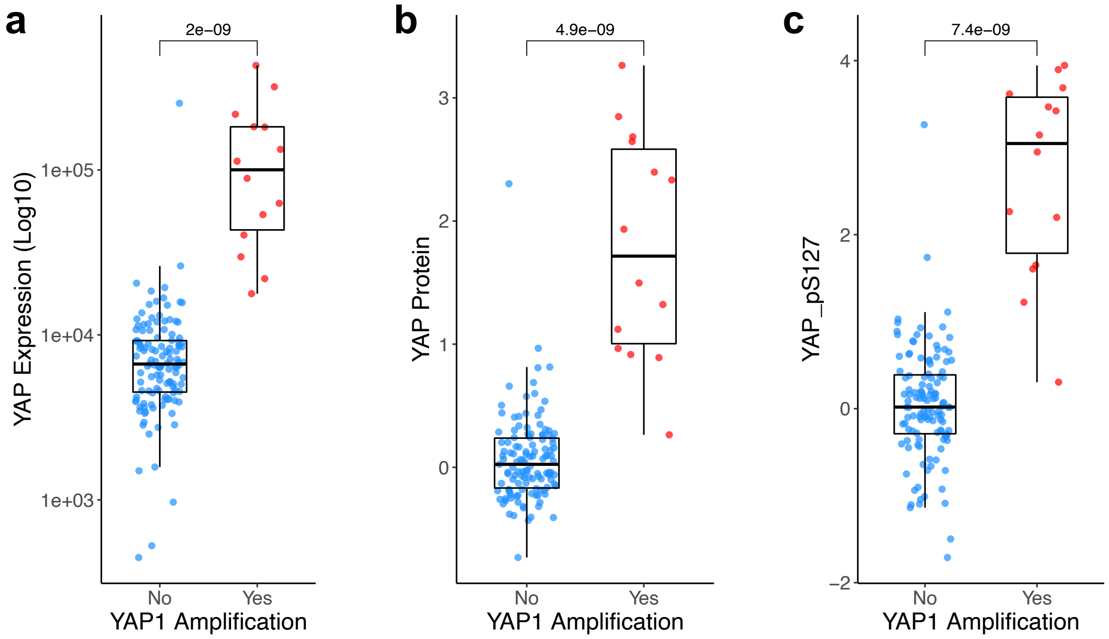

Supplementary Figure 8 Increased levels of YAP in tumours with YAP1 amplification. YAP1 expression **(a)**, and YAP protein levels **(b)** unphosphorylated and **c)** phosphorylated are higher in tumours that contain YAP1 amplifications for TCGA patients for which expression and reverse phase protein array (RPPA) data was available (n = 137 patients). Blue points represent tumours without YAP1 amplification and red points represent tumours with YAP1 amplification. The upper line in a box plot represents the upper quartile, the second line the median and the lowest line the lower quartile. The whisker above the box is drawn to the highest point within 1.5x the interquartile range (IQR), the whisker below the box is drawn to the lowest point within 1.5x the IQR. P values are from two-sided Wilcoxon's rank sum test. Source data is provided as a Source Data file.

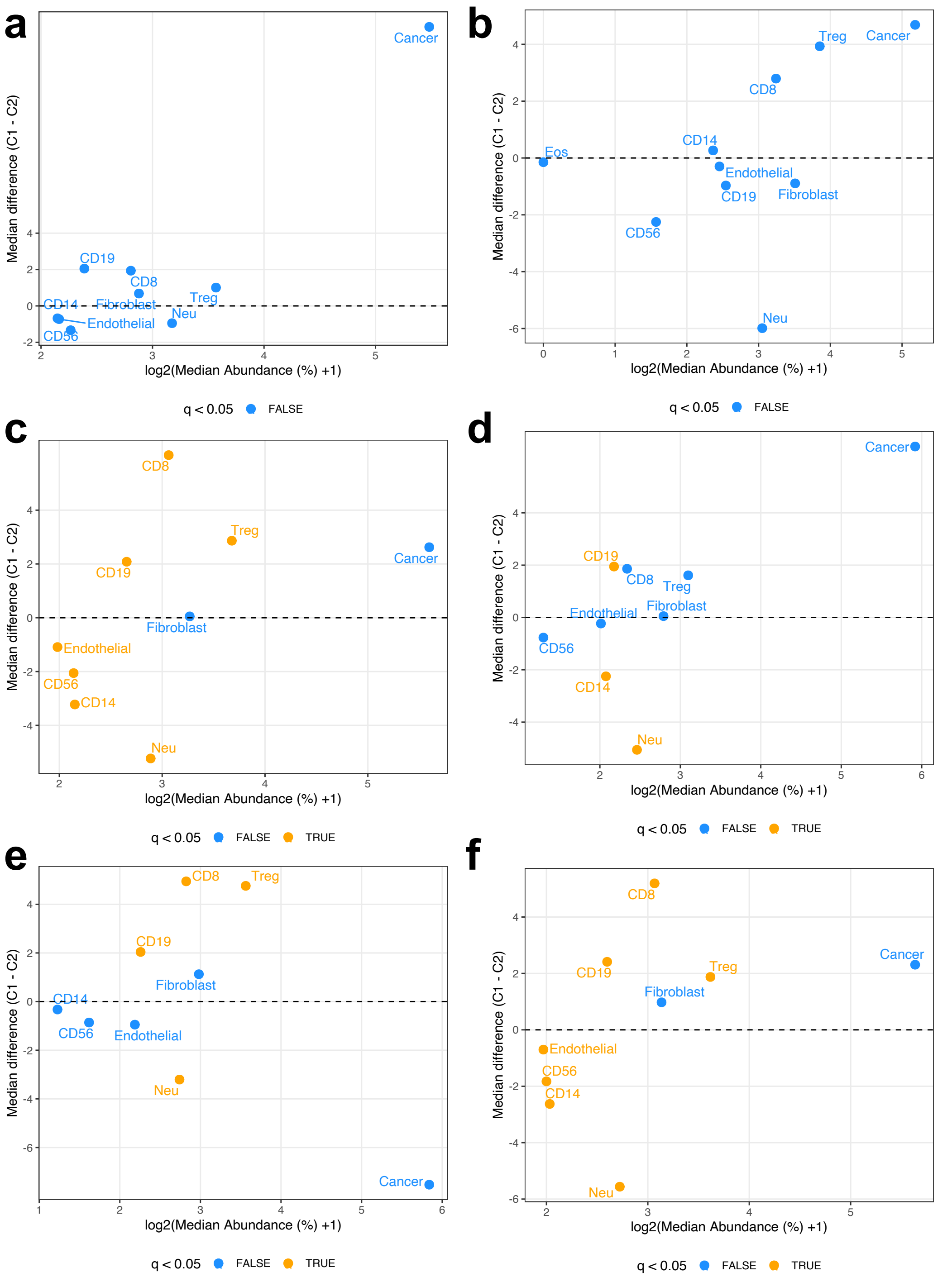

Supplementary Figure 9 Differences in immune microenvironment between SCC subgroups in individual cohorts. Median abundances (x-axis) and median differences (%, y-axis) for different cell types estimated using MethylCIBERSORT, with significant differences in orange for cohorts from **a)** Bergen, **b)** Innsbruck, **c)** Oslo and **d)** Uganda. These differences are also observed when analysis was restricted to HPV16 only for TCGA **(e)** and combined validation **(f)** cohorts. q values represent Benjamini-Hochberg corrected p values from Wilcoxon's rank sum test. Source data is provided as a Source Data file.

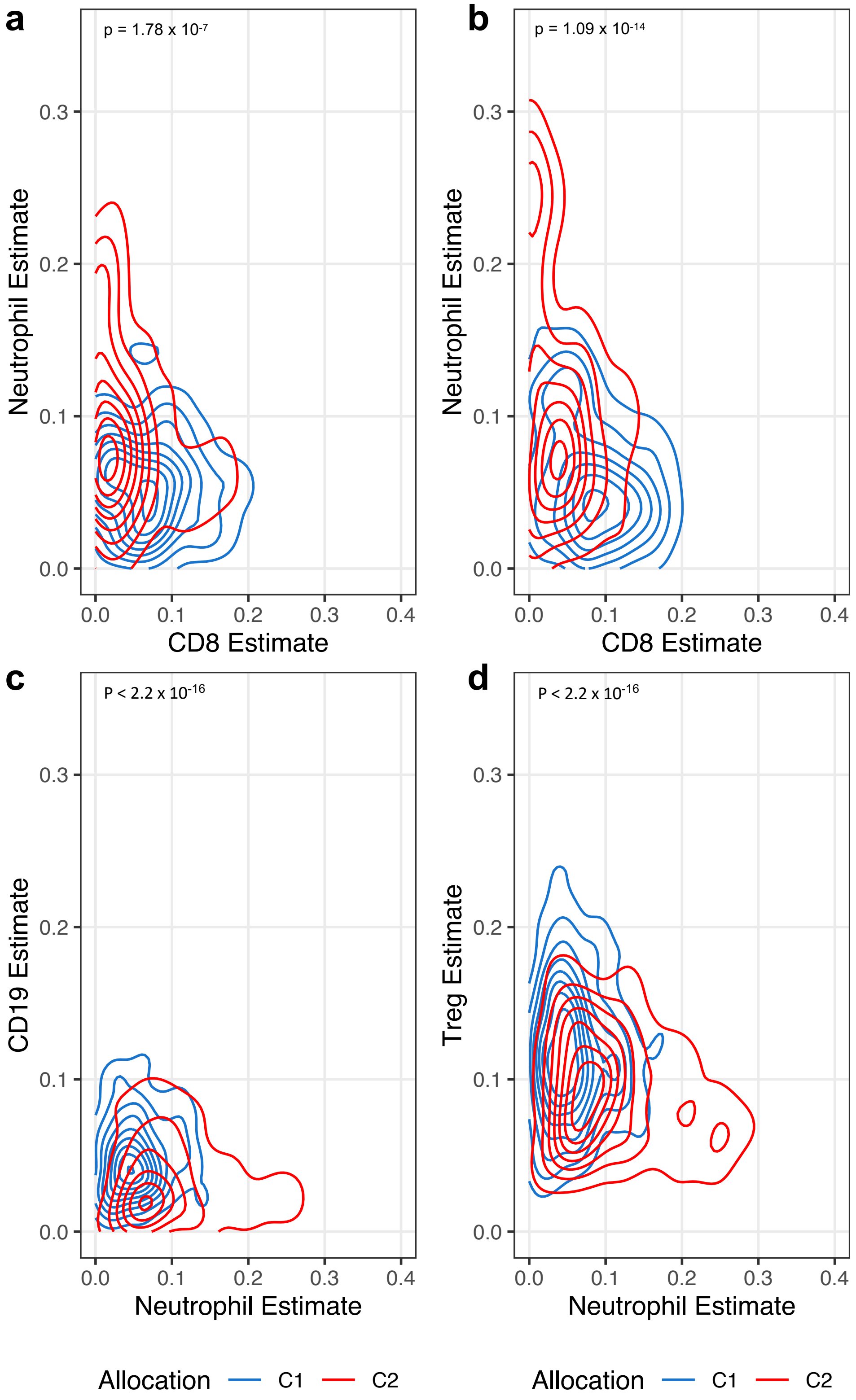

Supplementary Figure 10 Immune cell ratios by cluster using MethylCIBERSORT estimates. C2 tumours display increased neutrophil:CTL ratios as estimated using MethylCIBERSORT for **a)** TCGA discovery cohort and **b)** combined validation cohorts. Increased neutrophil:CD19 **(c)** and neutrophil:Treg ratios **(d)** were also seen when all cohort data was combined. P values are from two-sided Wilcoxon's rank sum test. Source data is provided as a Source Data file.

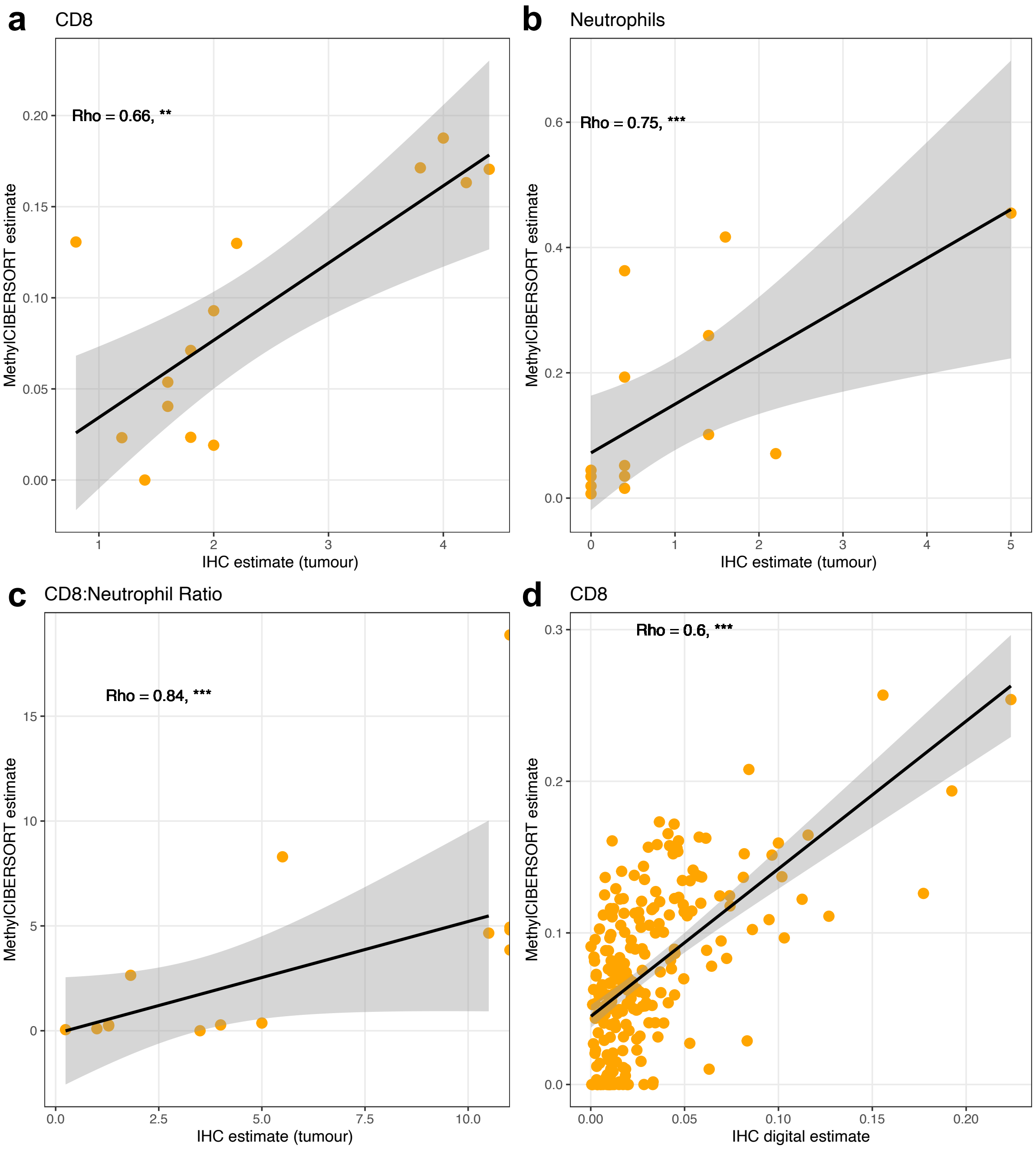

Supplementary Figure 11 Comparison of MethylCIBERSORT estimates and immunohistochemistry(IHC)-based scoring. Spearman correlation (Rho) between MethylCIBERSORT estimates and IHC-based scoring for **a)** CD8+ T-cells, **b)** neutrophils (MPO+), **c)** CD8+ T-cell:neutrophil ratio in 14 SCCs from the Innsbruck validation cohort and **d)** CD8+ T-cells for 229 SCCs from the Oslo validation cohort. Trendlines are derived from linear modelling, shaded areas represent 95% CI of trendlines. Spearman rank-order correlation ** $p < 0.05$ *** $p < 0.01$. Confidence bands (grey) represent the upper and lower 95% confidence interval limits. Source data is provided as a Source Data file.

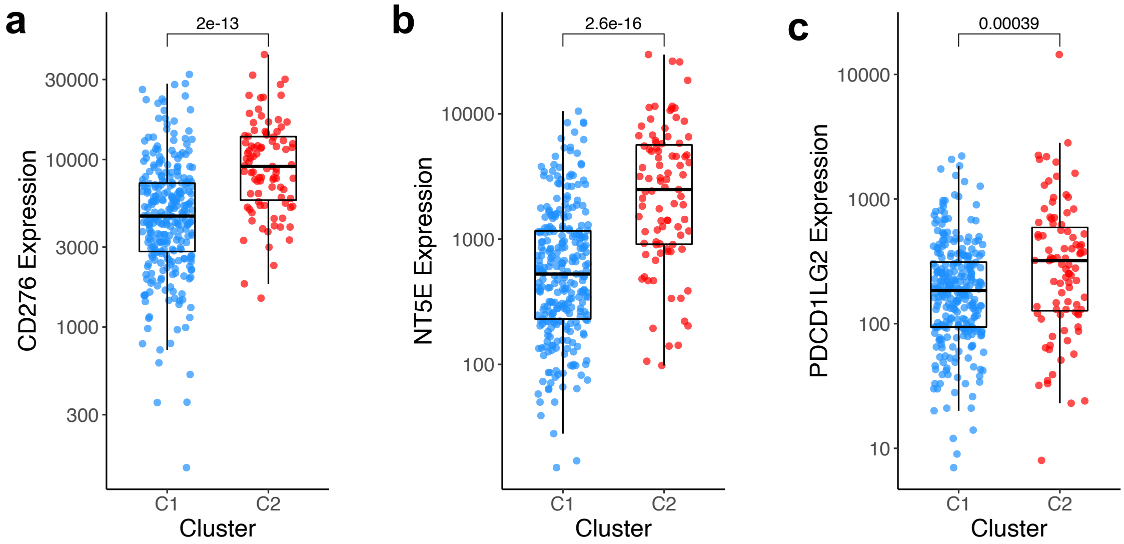

Supplementary Figure 12 Upregulation of immune checkpoint genes in C2 SCCs. Analysis performed with RNA-seq data from TCGA (n = 236), Bergen (n = 37) and Ugandan (n = 94) cohorts (total n = 367) shows upregulation of **a)** *B7-H3* (*CD276*), **b)** *NT5E* (*CD73*) and **c)** *PD-L2* (*PDCD1LG2*) in poor prognosis C2 tumours. Blue points represent C1 tumours and red points represent C2 tumours. The upper line in a box plot represents the upper quartile, the second line the median and the lowest line the lower quartile. The whisker above the box is drawn to the highest point within 1.5x the interquartile range (IQR), the whisker below the box is drawn to the lowest point within 1.5x the IQR. The P value is from two-sided Wilcoxon's rank sum test. Source data is provided as a Source Data file.
