# Supplementary material for: Integrated analysis of cervical squamous cell carcinoma cohorts from three continents reveals conserved subtypes of prognostic significance
Source: Nat Commun. 2022 Oct 7;13:5818. doi: 10.1038/s41467-022-33544-x (PMC9547055; doi:10.1038/s41467-022-33544-x)
Supplement: Supplementary file 3 — Reporting Summary [file 41467_2022_33544_MOESM3_ESM.pdf]

nature portfolio

Corresponding author(s): Tim Fenton
Andrew Feber
Kerry Chester

Last updated by author(s): Sep 2, 2022

Reporting Summary

Nature Portfolio wishes to improve the reproducibility of the work that we publish. This form provides structure for consistency and transparency in reporting. For further information on Nature Portfolio policies, see our Editorial Policies and the Editorial Policy Checklist.

Statistics

For all statistical analyses, confirm that the following items are present in the figure legend, table legend, main text, or Methods section.

n/a	Confirmed	
☐	☒	The exact sample size (n) for each experimental group/condition, given as a discrete number and unit of measurement
☒	☐	A statement on whether measurements were taken from distinct samples or whether the same sample was measured repeatedly
☐	☒	The statistical test(s) used AND whether they are one- or two-sided<br>*Only common tests should be described solely by name; describe more complex techniques in the Methods section.*
☐	☒	A description of all covariates tested
☐	☒	A description of any assumptions or corrections, such as tests of normality and adjustment for multiple comparisons
☐	☒	A full description of the statistical parameters including central tendency (e.g. means) or other basic estimates (e.g. regression coefficient) AND variation (e.g. standard deviation) or associated estimates of uncertainty (e.g. confidence intervals)
☐	☒	For null hypothesis testing, the test statistic (e.g. F, t, r) with confidence intervals, effect sizes, degrees of freedom and P value noted<br>*Give P values as exact values whenever suitable.*
☒	☐	For Bayesian analysis, information on the choice of priors and Markov chain Monte Carlo settings
☒	☐	For hierarchical and complex designs, identification of the appropriate level for tests and full reporting of outcomes
☐	☒	Estimates of effect sizes (e.g. Cohen's d, Pearson's r), indicating how they were calculated

Our web collection on statistics for biologists contains articles on many of the points above.

Software and code

Policy information about availability of computer code

Data collection	SRA Toolkit (http://ncbi.github.io/sra-tools/)
Data analysis	Supporting R packages used included dplyr v1.0.9, matrixStats v0.52.2, NMF v0.24.0, DescTools v0.99.45, ggplot2 v3.3.6, reshape v0.8.9, GGally v2.1.2, Coin v1.4-2, ccaPP v0.3.3, magrittr v2.0.3, DESeq2, reshape2 v1.4.4, multtest v2.24.0, amap v0.8-18, dynpred v0.1.2, gridExtra v2.3, stringr v1.4.1, tidyr v1.2.0, broom v1.0.0, ggrepel v0.9.1, gtsummary v1.6.1, TxDb.Hsapiens.UCSC.hg19.knownGene v3.1.2, tximport v1.3.9, EnsDb.Hsapiens.v75 v2.99.0, mutsignatures v2.1.1, BSgenome.Hsapiens.UCSC.hg19 v1.3.1000, and dependencies.<br>Other software: CIBERSORT (cibersort.stanford.edu); MethylCIBERSORT 0.2.1 (doi: 10.5281/zenodo.1298968); GISTIC2.0 (https://omictools.com/gistic-tool); Ingenuity Pathway Analysis (Qiagen)

For manuscripts utilizing custom algorithms or software that are central to the research but not yet described in published literature, software must be made available to editors and reviewers. We strongly encourage code deposition in a community repository (e.g. GitHub). See the Nature Portfolio guidelines for submitting code & software for further information.

Data

Policy information about availability of data

All manuscripts must include a data availability statement. This statement should provide the following information, where applicable:

- Accession codes, unique identifiers, or web links for publicly available datasets
- A description of any restrictions on data availability
- For clinical datasets or third party data, please ensure that the statement adheres to our policy

All data used in the study are publicly available. The DNA methylation (Illumina Infinium 450k array) data generated in this study have been deposited in the Gene Expression Omnibus under accession code GSE211668 (https://www.ncbi.nlm.nih.gov/geo/query/acc.cgi?acc=GSE211668). TCGA CESC DNA methylation (Illumina Infinium 450k array) and RNAseq data are available from the TCGA data portal. TCGA mutation data are available from the MC3 project on SAGE Synapse (syn7214402). RNAseq data for the Uganda cohort are available from the TCGA data portal and DNA methylation (Illumina Infinium EPIC array) and mutation data from National Cancer Institute's Genome Data Commons Publication Page at https://gdc.cancer.gov/about-data/publications/CGCI-HTMCP-CC-2020. DNA methylation (Illumina Infinium 450k array) and gene expression (Illumina HumanHT-12 V4.0 expression beadchip) data from the Oslo cohort are available from the Gene Expression Omnibus (GSE68339). RNAseq data were obtained for the Bergen cohort from dbGaP (phs000600/DS-CA-MDS 'Genomic Sequencing of Cervical Cancers') under the authorisation of project #14589 "Investigating the mechanisms by which viruses and carcinogens contribute to cancer development". Source data are provided with this paper.

Human research participants

Policy information about studies involving human research participants and Sex and Gender in Research.

Reporting on sex and gender	N/A - entire cohort female (cervical cancer).
Population characteristics	Detailed sample-by-sample information on all patients is provided in Supplementary Table S1 (TCGA patients) and Supplementary Table S6 (validation cohort patients). Key clinicopathologic characteristics of each cohort are also summarised in Table 1.
Recruitment	All patients gave written, informed consent before inclusion. Samples from Bergen were collected in a population-based setting from patients treated at the Department of Obstetrics and Gynaecology, Haukeland University Hospital, Bergen, Norway, from May 2001 to May 2011. Samples from Innsbruck were collected and processed at the Department of Obstetrics and Gynaecology of the Medical University of Innsbruck. Samples from Oslo (n = 268) were collected from patients participating in a previously published prospective clinical study.
Ethics oversight	The Bergen study was approved by the Regional Committee for Medical Research Ethics in Western Norway (REK 2009/2315, 2014/1907 and 2018/591).<br>The Innsbruck study was reviewed and approved by the Ethics committee of the Medical University of Innsbruck (reference number: AN2016-0051 360/4.3; 374/5.4: 'Biobank study: Validation of a DNA-methylation based signature in cervical cancer').<br>The Oslo study was approved by the Regional Committee for Medical Research Ethics in southern Norway (REK no. S-01129).

Note that full information on the approval of the study protocol must also be provided in the manuscript.

Field-specific reporting

Please select the one below that is the best fit for your research. If you are not sure, read the appropriate sections before making your selection.

☒ Life sciences ☐ Behavioural & social sciences ☐ Ecological, evolutionary & environmental sciences

For a reference copy of the document with all sections, see nature.com/documents/nr-reporting-summary-flat.pdf

Life sciences study design

All studies must disclose on these points even when the disclosure is negative.

Sample size	All analyses were performed using the maximum number of samples for which the necessary molecular and clinicopathologic data were available.
Data exclusions	Samples were only excluded from analyses if necessary data were not available and this has been stated in the manuscript.
Replication	The study is based on a discovery cohort (TCGA, n = 236), in which we made observations that we then demonstrated were reproducible in independent validation cohorts (Europe combined, n = 313, Uganda, n = 94).
Randomization	Randomization was not relevant to the analyses performed in this study as no experiments were performed in which patients were separated into groups (this was not an intervention study).

Blinding

All immunohistochemistry staining and scoring (used to validate DNA methylation-based estimates of immune cell infiltrates in tumour samples) was performed blinded to the DNA methylation based estimates and the cluster (C1 or C2) to which the tumour sample had been allocated. Inspection of H&E images of TCGA samples from the Digital Slide Archive (https://cancer.digitalslidearchive.org) was performed blinded to TCGA-assigned histology. Investigators were blinded to cluster allocation during all data collection and analysis, as cluster allocation was performed post-data collection and analysis.

Reporting for specific materials, systems and methods

We require information from authors about some types of materials, experimental systems and methods used in many studies. Here, indicate whether each material, system or method listed is relevant to your study. If you are not sure if a list item applies to your research, read the appropriate section before selecting a response.

Materials & experimental systems

n/a	Involved in the study
☐	☒ Antibodies
☒	☐ Eukaryotic cell lines
☒	☐ Palaeontology and archaeology
☒	☐ Animals and other organisms
☒	☐ Clinical data
☒	☐ Dual use research of concern

Methods

n/a	Involved in the study
☒	☐ ChIP-seq
☒	☐ Flow cytometry
☒	☐ MRI-based neuroimaging

Antibodies

Antibodies used

Anti-CD8 (mouse monoclonal 4B11, Leica Biosystems PA0183); anti-MPO (rabbit polyclonal, Agilent A039829-2)

Validation

Anti-CD8 (from the manufacturer's website): For in vitro diagnostic use. NCL-L-CD8-4B11 is intended for the qualitative identification by light microscopy of human CD8 antigen in paraffin sections.

From the manufacturer's website ('Expected Results'):

Normal Tissues
Clone 4B11 detects the CD8 antigen on the cell surface of the cytotoxic sub-population of T-cells in thymus, spleen, lymph nodes and tonsil. (Total number of normal cases evaluated = 44).
Abnormal Tissues
Clone 4B11 stained 3/4 angioimmunoblastic T-cell lymphomas. Except for reactive T-cells, no staining was detected in diffuse large B-cell lymphomas (0/108), chronic lymphocytic lymphomas (0/12), follicular lymphomas (0/11), Hodgkin's disease (0/11), mantle cell lymphomas (0/7), T-cell anaplastic large cell lymphomas (0/7), T/NK cell lymphomas (0/3), a B-cell acute lymphoblastic lymphoma (0/1),
a primitive B/T cell acute lymphoblastic lymphoma (0/1), a peripheral T-cell lymphoma (0/1), a T-cell lymphoma (0/1), a marginal zone
lymphoma (0/1), tumors of the thyroid (0/4), lung tumors (0/4), ovarian tumors (0/4), liver tumors (0/4), brain tumors (0/2), esophageal
tumors (0/2), breast tumors (0/2), gastric tumors (0/2), soft tissue tumors (0/2), tumors of the tongue (0/2), metastatic tumors of unknown
origin (0/2), kidney tumors (0/2), cervical tumors (0/2), testicular tumors (0/2), tumors of the colon (0/2), rectal tumors (0/2), skin tumors
(0/2), a tumor of the larynx (0/1) and a tumor of the thymus (0/1). (Total number of tumor cases evaluated = 212)
From our methods section: All CD8 series included positive controls. Negative controls included substitution of the monoclonal antibody with mouse myeloma protein of the same subclass and concentration as the monoclonal antibody. All controls gave satisfactory results.

Anti-MPO (from the manufacturer's website): The antibody reacts with human myeloperoxidase. In crossed immunoeletrophoresis using 12.5 uL antibody per cm2 gel area against 2 uL myeloperoxidase only one precipitate corresponding to myeloperoxidase appears. Staining: specific staining for peroxidase. With 2 uL of human plasma, no precipitate appears. Staining: Coomassie Brilliant Blue. In formalin-fixed, paraffin-embedded human tonsil, and when using a sensitive streptavidin-biotin-based visualization system, the antibody labels only rare neutrophil granulocytes.
