# Supplementary material for: Integrated analysis of cervical squamous cell carcinoma cohorts from three continents reveals conserved subtypes of prognostic significance
Source: Nat Commun. 2022 Oct 7;13:5818. doi: 10.1038/s41467-022-33544-x (PMC9547055; doi:10.1038/s41467-022-33544-x)
Supplement: Supplementary file 4 — Description of Additional Supplementary Files [file 41467_2022_33544_MOESM4_ESM.docx]

**Description of Additional Supplementary Files**

File Name:

Supplementary Data 1

Description: Clinical and pathologic characteristics of TCGA squamous cervical cancer cohort samples

File Name: Supplementary Data 2

Description: Top 10% most variable genes in TCGA squamous cervical cancer cohort

File Name: Supplementary Data 3

Description: 938 Differentially expressed genes between TCGA squamous cervical cancer clusters C1 and C2

File Name: Supplementary Data 4

Description: Survival uni- and multivariate analysis for HPV16+ patients in squamous cervical cancer cohorts

File Name: Supplementary Data 5

Description: 129 MVP signature probes (European validation cohorts)

File Name: Supplementary Data 6

Description: Combined validation cohort cluster allocation

File Name: Supplementary Data 7

Description: Breakdown of tumour stage in C1 and C2 cluster by percentage

File Name: Supplementary Data 8

Description: Clusters and EMT scores for TCGA squamous cervical cancer samples

File Name: Supplementary Data 9

Description: Significantly mutated genes using dNdSCV analysis and combining cohorts

File Name: Supplementary Data 10

Description: Mutation frequency in SMGs observed in previous studies

File Name: Supplementary Data 11

Description: Gene set enrichment analysis of C2 gene expression signature genes using Metascape

File Name: Supplementary Data 12

Description: Histology of TCGA samples by pathologist blinded to cluster allocation

File Name: Supplementary Data 13

Description: Paramaters for TSNE multidimensional visualisation analyses

File Name: Supplementary Data 14

Description: 116 MVP signature probes (Ugandan validation cohort)
